# Candidate genes and pathways downstream of PAX8 involved in ovarian high-grade serous carcinoma

**DOI:** 10.18632/oncotarget.9740

**Published:** 2016-05-31

**Authors:** Tiziana de Cristofaro, Tina Di Palma, Amata Amy Soriano, Antonella Monticelli, Ornella Affinito, Sergio Cocozza, Mariastella Zannini

**Affiliations:** ^1^ IEOS, Institute of Experimental Endocrinology and Oncology “G. Salvatore”, National Research Council, Naples, Italy; ^2^ Department of Molecular Medicine and Medical Biotechnology, University of Naples Federico II, Naples, Italy

**Keywords:** PAX8, ovarian cancer, fallopian tube secretory epithelial cells, RNA-seq, transcriptional networks

## Abstract

Understanding the biology and molecular pathogenesis of ovarian epithelial cancer (EOC) is key to developing improved diagnostic and prognostic indicators and effective therapies. Although research has traditionally focused on the hypothesis that high-grade serous carcinoma (HGSC) arises from the ovarian surface epithelium (OSE), recent studies suggest that additional sites of origin exist and a substantial proportion of cases may arise from precursor lesions located in the Fallopian tubal epithelium (FTE). In FTE cells, the transcription factor PAX8 is a marker of the secretory cell lineage and its expression is retained in 96% of EOC. We have recently reported that PAX8 is involved in the tumorigenic phenotype of ovarian cancer cells. In this study, to uncover genes and pathways downstream of PAX8 involved in ovarian carcinoma we have determined the molecular profiles of ovarian cancer cells and in parallel of Fallopian tube epithelial cells by means of a silencing approach followed by an RNA-seq analysis. Interestingly, we highlighted the involvement of pathways like WNT signaling, epithelial-mesenchymal transition, p53 and apoptosis. We believe that our analysis has led to the identification of candidate genes and pathways regulated by PAX8 that could be additional targets for the therapy of ovarian carcinoma.

## INTRODUCTION

Ovarian cancer, like other cancers, is a spectrum of diseases and not a single disease entity [1]. Malignant surface epithelial tumors (carcinomas) are the most common ovarian cancers, accounting for 90% of the cases. These tumors are divided in four major histotypes: serous, mucinous, endometrioid and clear cell. Of these, high-grade serous carcinoma (HGSC) is the most common and lethal histotype, associated with abnormalities of *BRCA1, BRCA2* and p53 [2]. This cancer is extremely genetic unstable and heterogenous; it is rarely detected while confined to the ovary and in about 90% of the cases it is diagnosed when women have already intra-abdominal spread. Although research has traditionally focused on the hypothesis that HGSC arises from the ovarian surface epithelium (OSE), it has been recently suggested that it may arise from the Fallopian tube fimbria. In particular, the serous tubal intraepithelial carcinomas (STICs), which are identified in the distal end (fimbria) of the Fallopian tube and arise from p53 mutations, have been indicated as the primary lesions that evolve through subsequent oncogenic events into HGSC [3]. This hypothesis is further supported by genetically engineered murine models that mimic the transformation from Fallopian tubal secretory epithelial cells to HGSC [4].

The transcription factor PAX8 is a marker of the Fallopian tube secretory cell lineage and its expression is retained in 96% of the serous ovarian carcinomas, in 89% of the endometrioid and 100% of the clear cell carcinomas, whilst it is not detected in the mucinous carcinoma [5]. PAX8 is a member of the PAX (PAired boX) gene family, genes tightly regulated in both temporal and spatial expression patterns [6]. PAX8 is crucial for the organogenesis of the thyroid gland, kidney, nervous system and Mullerian system [7, 8]. In the adult, PAX8 is expressed in the thyroid gland, in the renal excretory system and in tissues derived from the Mullerian ducts [9, 10]. Our recent results demonstrated that PAX8 plays a critical role in cell cycle progression and cell survival of differentiated epithelial cells [11], reinforcing the involvement of this transcription factor in different biological processes. In tumors, PAX8 is involved in the progression of follicular thyroid carcinomas [12] and it is overexpressed in the majority of gliomas, Wilms tumors and well-differentiated pancreatic neuroendocrine tumors [10, 13, 14]. In the scenario of ovarian cancer, PAX8 is the currently available most important marker [15] being a useful IHC target for the diagnosis of Mullerian tumors [16]. Despite PAX8 is normally expressed in Fallopian tube secretory cells, namely the cell of origin of HGSC, and is mainly considered a marker of tumor origin, we have recently reported its pivotal function in the tumorigenic phenotype of ovarian cancer cells. In particular, we showed that PAX8 plays a critical role in the migration, invasion and tumorigenic ability of ovarian cancer cells. In our published study, PAX8 silencing strongly suppressed anchorage-independent growth *in vitro* and significantly inhibited tumorigenesis *in vivo* in a nude mouse xenograft model [17]. In addition, the Cancer Genome Atlas (TGCA) Project indicated PAX8 as a survival gene essential for the proliferation of ovarian cancer cells [5]. Overall, PAX8 belongs to a class of lineage-survival genes that are required for both normal development of specific tissues and for cancer cell proliferation/survival.

On the basis of such evidences, it is conceivable that PAX8 is intimately involved in the progression of ovarian carcinomas. However, its function in Fallopian tube secretory epithelial cells, as well as the entire molecular network that allows PAX8 to regulate cellular processes in these cells and in ovarian cancer cells, is still an unexplored field. To improve our knowledge of the complex role of this transcription factor, we thought that it would be beneficial to uncover the downstream gene network governed by this transcriptional factor both in Fallopian tubal epithelium and ovarian cancer cells. Hence, the aims of our study were (1) to compare the transcriptome of normal Fallopian tube secretory epithelial cells with that of ovarian cancer cells in order to uncover genes and pathways modified during the transformation process; (2) to investigate PAX8 downstream gene regulatory network in physiological and pathological conditions to identify genes and pathways regulated by PAX8 that could be additional targets for the therapy of ovarian carcinoma.

## RESULTS

### Identification of genes differentially expressed between FT194 and SKOV-3 cells

Primary questions in the field of ovarian cancer biology concern its developmental cell of origin. The epithelial cells covering the ovaries (OSE) have historically been considered the site of origin of all ovarian cancers but recent evidence suggests that high-grade serous carcinoma (HGSC) originates from the Fallopian tube (FT) epithelium or the tuboperitoneal junction rather than from the OSE [4, 18].

To identify genes differentially expressed in the Fallopian tubal epithelium and in ovarian carcinoma and to disclose some of the pathways that might contribute to the carcinoma formation, an RNA sequencing analysis was performed on FT194 Fallopian tube secretory epithelial cells and SKOV-3 ovarian adenocarcinoma cells and a total of 12628 genes were mapped to the reference human genome (Supplementary Table S1). Of these, 7451 genes were differentially expressed in the two cell types at significant level (FDR-adjusted *p*-value ≤ 0.05) (Supplementary Table S2): 3553 genes resulted more expressed in SKOV-3 cells than in FT194 cells and 3778 genes were found less expressed in SKOV-3 cells with respect to FT194 cells. Interestingly, 38 genes were exclusively expressed in SKOV-3 cells, while 82 genes were present only in FT194 cells (Supplementary Table S2).

**Figure 1:**
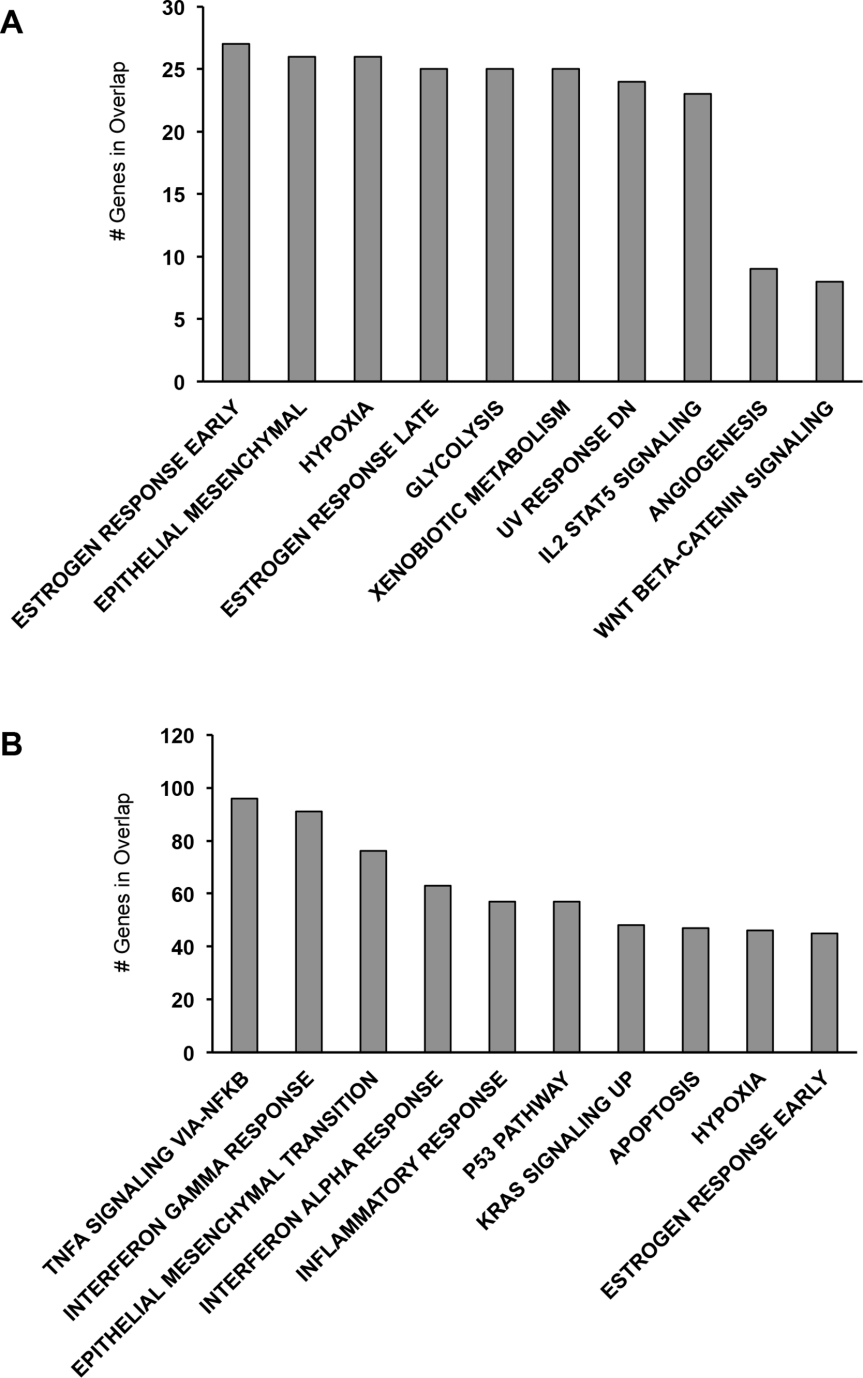
MsigDB analysis of the most relevant genes differentially expressed between FT194 and SKOV-3 cells (**A**) MsigDB for genes upregulated ≥ 2 fold in SKOV-3 cells. (**B**) MsigDB for genes downregulated ≥ 2 fold in SKOV-3 cells.

To date, despite the identification of a number of key mutations in p53 and BRCA1/2 genes, the complexity of the molecular pathway(s) underlying epithelial ovarian cancer has not been yet fully elucidated. To focus our attention on genes showing the greatest expression differences between FT194 and SKOV-3 cells, we applied a 2 fold-change cutoff and we searched for enrichment of specific gene sets at the Molecular Signatures Database (MsigDB). In particular, we explored the hallmark gene sets that summarize and represent specific well-defined biological states or processes. As shown in Figure 1A, genes up-regulated ≥ 2 fold in SKOV-3 cells present a significant enrichment for pathways such as estrogen response, epithelial mesenchymal transition, response to UV DNA damage, angiogenesis and Wnt/β-catenin signaling. There are numerous evidences, both *in vitro* and *in vivo* studies, that estrogens might regulate ovarian carcinogenesis [19]. It is well known that hormones stimulate the proliferation of the cells, meaning that they might function as carcinogens. For example, in ovarian cancer cells 17β-estradiol (E2) increases the ROS and NO production that participates in cancer progression [20]. In addition, aberrant Wnt/β-catenin signaling is implicated in several cancers including epithelial ovarian cancer [21, 22] and it represents one of the pathway involved in epithelial-mesenchymal transition (EMT) responsible for cancer cell dissemination and metastasis formation [23]. Stressful conditions such as UV radiations, hypoxia, and alterations associated with changes in cell phenotype that include EMT as well as angiogenesis represent main processes that provide carcinogenesis. At the same time, genes down-regulated ≥ 2 fold in SKOV-3 cells (Figure 1B) are involved in pathways as TNF-α signaling, IFN-α and IFN-γ responses and inflammatory response. TNF-α, IFN-α and IFN-γ are pleiotropic cytokines with diverse physiological functions such as activation of macrophages, induction of immune response and apoptosis and inhibition of cell proliferation. It has been reported that in ovarian cancer the above-mentioned cytokines exert an anti-proliferative activity and induce apoptosis [24, 25]. Eventually, if the immune response might contribute to the tumorigenic process, it may be also helpful in preventing the tumor properties.

Among the 38 genes exclusively expressed in SKOV-3 cells (Supplementary Table S2), ALDH1A1 [26], GABRA3 [27], FOLR1 [28], DPYSL5 [29], CGB8 [30], C8orf4 [31] and MAGEB2 [32] could be intriguing for the development and maintenance of the neoplastic phenotype. Similarly, among the 82 genes expressed only in FT194 cells (Supplementary Table S2) CDKN2A [33], MT1G [34], GPX7 [35], HCK [36], ZBTB16 [37] could be looked at as interesting genes being tumor suppressor epigenetically silenced in cancer cells.

### PAX8 downstream target genes in FT194 and SKOV-3 cells

As already mentioned, in the Fallopian tube epithelium the transcription factor PAX8 is a marker of the secretory cell lineage. Our RNA sequencing analysis performed on FT194 Fallopian tube secretory epithelial cells and SKOV-3 ovarian adenocarcinoma cells confirmed that PAX8 is expressed at similar levels in these two cell types (Supplementary Table S2), supporting the hypothesis that PAX8 is not overexpressed in epithelial ovarian carcinoma but rather that its expression is conserved from the suggested cell of origin [38]. Recently, we have provided the first evidence of a clear involvement of PAX8 in the *in vivo* tumorigenesis of ovarian cancer cells [17], reinforcing the role of *PAX* genes in cancer through their effects on apoptosis resistance, tumor cell proliferation and migration, and repression of terminal differentiation.

On the basis of our previous data and in order to clarify PAX8 contribution to ovarian cancer through the identification of its downstream gene regulatory network, we have transiently knocked-down PAX8 expression in both FT194 and SKOV-3 cells. We chose to inhibit PAX8 expression also in the FT194 cell line because to date its role in the physiological contest of the Fallopian tube secretory cell has not been investigated. Moreover, we reasoned that genes affected by PAX8 silencing in both cell lines could be looked at as PAX8 targets with greater confidence.

Three independent silencing experiments were performed and analyzed by RNA-seq unraveling a total of 467 genes modulated by PAX8 expression in SKOV-3 and FT194 cells (Supplementary Table S3 and Supplementary Table S4). The reduced expression of PAX8 in the samples was confirmed by the RNA-seq findings, which showed an 80% decrease (FDR-adjusted *p*-value ≤ 0.05) (Supplementary Table S3 and Supplementary Table S4).

In SKOV-3 cells, 301 genes were significantly modulated by PAX8 silencing after correction for multiple testing (FDR-adjusted *p*-value ≤ 0.05) of which 214 were downregulated while 87 resulted upregulated (Supplementary Table S3). As shown in Table 1, among the most downregulated genes based on FDR there are *TUBB2B, PPME1, ATP8A1, USP2, BLCAP, DIAPH1, FGF18* and *KIF12*.

**Table 1:** Differentially expressed genes that are significantly modulated after PAX8 silencing in SKOV– 3 cells

	Gene Name	NCBI gene ID	FPKM1	FPKM2	Log_2_ fold change	FDR *q* value	Fold change
1	ANKRD34B	340120	0,517453	2,34955	2,18288	0,00400937	4,54059
2	PAX8	7849	8,65929	30,224	1,80338	0,00400937	3,49037
3	TUBB2B	347733	0,483395	1,54683	1,67804	0,015208	3,19993
4	PPME1	51400	11,285	34,0561	1,59351	0,00400937	3,01783
5	LMLN	89782	0,558739	1,61854	1,53445	0,00400937	2,89678
6	H2AFY2	55506	6,68489	19,0865	1,51358	0,00400937	2,85518
7	PHTF2	57157	3,23708	8,76508	1,43707	0,00400937	2,70770
8	DCAF12L1	139170	1,66899	4,506	1,43287	0,00400937	2,69983
9	GRIN2B	2904	0,727982	1,94915	1,42087	0,0100329	2,67747
10	EPRS	2058	18,5003	47,5384	1,36154	0,00400937	2,56959
11	PPAP2B	8613	5,23887	13,3333	1,3477	0,00400937	2,54506
12	ATP8A1	10396	0,686376	1,74582	1,34683	0,00400937	2,54353
13	USP2	9099	0,582276	1,43193	1,29819	0,0127719	2,45920
14	BLCAP	10904	9,66632	23,4671	1,2796	0,00400937	2,42771
15	FGF18	8817	2,82984	6,85593	1,27663	0,00400937	2,42272
16	DIAPH1	1729	5,21853	12,5088	1,26123	0,00400937	2,39700
17	KIF12	113220	2,32013	5,50046	1,24535	0,00400937	2,37076
18	C10orf46	143384	14,0943	33,1881	1,23555	0,00400937	2,35471
19	ANXA2P2	304	1,92906	4,516	1,22715	0,00400937	2,34104
20	PSAP	5660	201,624	467,609	1,21364	0,00400937	2,31922
21	DIO2	1734	5,30475	1,69721	– 1,64412	0,00400937	3,12557
22	ROS1	6098	2,97713	1,06039	– 1,48933	0,00400937	2,80759
23	P4HA3	283208	1,91889	0,746284	– 1,36248	0,00400937	2,57127
24	PTPMT1	114971	14,4328	5,88494	– 1,29426	0,0196014	2,45251
25	MET	4233	47,8695	20,5651	– 1,21891	0,00400937	2,32771
26	CCIN	881	1,24831	0,566005	– 1,14109	0,0361254	2,20548
27	ARTN	9048	5,77216	2,63816	– 1,12958	0,00400937	2,18795
28	LOC100507412	100507412	394,087	186,2	– 1,08166	0,00400937	2,11647
29	SERPINB5	5268	1,49223	0,722489	– 1,04642	0,0340659	2,06540
30	SERPINE1	5054	128,607	62,4787	– 1,04153	0,00400937	2,05841
31	DKK1	22943	85,9562	42,0833	– 1,03035	0,00400937	2,04252
32	SGK1	6446	18,4838	9,10546	– 1,02146	0,00400937	2,02997
33	FGF1	2246	1,67617	0,843698	– 0,990371	0,00400937	1,98670
34	BOD1	91272	27,4212	14,2379	– 0,945548	0,00400937	1,92592
35	CCND3	896	27,6423	14,3971	– 0,941101	0,00400937	1,91999
36	TGFB2	7042	13,4826	7,11507	– 0,922151	0,00400937	1,89494
37	CALU	813	57,1015	30,1837	– 0,919757	0,00400937	1,89180
38	ANKRD1	27063	10,4157	5,56174	– 0,905149	0,00400937	1,87274
39	CGB8	94115	11,0552	5,93536	– 0,897321	0,015208	1,86260
40	C6orf120	387263	18,0817	9,71162	– 0,896746	0,00400937	1,86186

Note: FPKM1 and FPKM2 indicate fragments per kilobase of exon per million mapped reads after and before PAX8 silencing, respectively. Genes from 1 to 20 are downregulated and genes from 21 to 40 are upregulated.

*TUBB2B* (tubulin beta 2B class IIb) may have important roles in tumor progression and chemoresistance [39]. *PPME1* is a protein phosphatase 2A (PP2A)-specific methylesterase that mediates the demethylation and inactivation of PP2A. This protein is a human tumor suppressor that accounts for the majority of cellular serine/threonine phosphatase activity [40]. *ATP8A1* is a member of P4-ATPase family with a role in the formation of membrane ruffles to promote cell migration [41]. *BLPAP* (Bladder cancer-associated protein) is a highly conserved protein among species displaying tissue-specific expression patterns, but also tissue-specific functions being both a tumor suppressor and a tumor promoting [42]. Recent data revealed that the blockade of DIAPH1-tubulin interaction might be a promising approach to inhibit one of the earliest steps in the metastatic cascade of colon cancer [43]. *FGF18* is a pleiotropic growth factor involved in skeletal growth and development [44, 45]. Deregulated FGF signaling may affect some oncogenic mechanisms and properties in various tumor types [46]. *KIF12* belongs to the kinesin superfamily of motor proteins that bind microtubules and mediate the intracellular transport of organelles and protein complexes [47].

Interestingly, in siPAX8-SKOV-3 cells among the most up-regulated genes based on FDR there are *DKK1* and *BOD-1* [48] (Table 1). DKK1 belongs to the *Dickkopf* gene family, which encodes secreted glycoproteins to control cell fate and neural patterning during embryonic development. It was first identified as an inhibitor of the Wnt signaling pathway. *BOD-1* is a member of the FAM44 protein family and is highly conserved throughout metazoans. Depletion of *BOD-1* in human cells causes severe biorientation defects [49].

In FT194 cells, 166 genes affected by PAX8 silencing reached genome-wide significance after correction for multiple testing (FDR-adjusted *p*-value ≤ 0.05) of which 119 were downregulated while 47 were upregulated (Supplementary Table S4). Among the most significantly downregulated genes there are: *FGF18, CDH6, CHRD, ZBED2, CNTN4, ANXA2* (Table 2). Very interestingly, *FGF18* results among the top 20 downregulated genes upon silencing of PAX8 in both SKOV-3 and in FT194 cells (Table 1 and Table 2).

**Table 2:** Differentially expressed genes that are significantly modulated after PAX8 silencing in FT194 cells

	Gene Name	NCBI gene ID	FPKM1	FPKM2	Log_2_ fold change	FDR *q* value	Fold change
1	CDH6	1004	0,904512	5,66947	2,648	0,00642248	6,26798
2	SNORA26	677810	12,5059	73,4428	2,55401	0,00642248	5,87264
3	FGF18	8817	2,9921	14,7197	2,29852	0,00642248	4,91953
4	CHRD	8646	1,14353	5,39776	2,23887	0,00642248	4,72027
5	KLHL14	57565	0,218892	0,999955	2,19164	0,00642248	4,56824
6	EPHB1	2047	0,17794	0,77373	2,12044	0,0162802	4,34827
7	ZBED2	79413	0,98563	4,13057	2,06722	0,00642248	4,19078
8	TLL2	7093	0,625627	2,60957	2,06043	0,00642248	4,17111
9	PAX8	7849	9,23106	33,8269	1,8736	0,00642248	3,66446
10	BAALC	79870	0,360111	1,28504	1,8353	0,0333357	3,56846
11	MAL	4118	7,14709	25,0391	1,80876	0,00642248	3,50341
12	DCDC2	51473	4,33336	14,9979	1,7912	0,00642248	3,46103
13	LOC643201	643201	1,32389	4,51658	1,77045	0,00642248	3,41160
14	ADAMTS14	140766	0,900768	3,0108	1,74092	0,00642248	3,34248
15	CNTN4	152330	0,800182	2,67321	1,74017	0,00642248	3,34075
16	ANXA2	302	572,227	1838,55	1,68391	0,00642248	3,21298
17	FAT2	2196	0,373409	1,18513	1,66622	0,00642248	3,17382
18	ANXA2P2	304	1,12435	3,55187	1,65948	0,0115711	3,15903
19	ROR1	4919	7,26579	22,3551	1,62141	0,00642248	3,07676
20	LMLN	89782	0,291647	0,876636	1,58775	0,0115711	3,00580
21	SERPINB2	5055	4,52656	1,12888	– 2,00352	0,00642248	4,00977
22	FPR1	2357	18,91	7,18704	– 1,39568	0,00642248	2,63113
23	PAPPA	5069	4,39506	1,94254	– 1,17794	0,00642248	2,26253
24	PTGS2	5743	14,4361	6,46952	– 1,15795	0,00642248	2,23140
25	NRK	203447	2,78453	1,25142	– 1,15387	0,00642248	2,22510
26	ST6GALNAC5	81849	10,3815	4,81544	– 1,10827	0,00642248	2,15587
27	THBD	7056	7,82686	3,66541	– 1,09446	0,00642248	2,13533
28	CCL20	6364	60,3995	30,2135	– 0,999342	0,0162802	1,99909
29	RHOB	388	44,0161	22,2603	– 0,983562	0,00642248	1,97734
30	F3	2152	132,89	67,4915	– 0,977451	0,00642248	1,96898
31	TGFB2	7042	53,4068	27,4917	– 0,958027	0,00642248	1,94265
32	ZCCHC2	54877	9,21038	4,7504	– 0,955212	0,00642248	1,93886
33	DCN	1634	20,2125	10,5292	– 0,940855	0,00642248	1,91967
34	MX2	4600	66,0911	34,8487	– 0,923353	0,00642248	1,89652
35	RSAD2	91543	72,3042	38,7711	– 0,899096	0,00642248	1,86490
36	MSRB3	253827	35,8124	19,2352	– 0,896709	0,00642248	1,86181
37	MET	4233	54,6992	30,1665	– 0,858572	0,00642248	1,81324
38	MX1	4599	264,614	148,11	– 0,837216	0,00642248	1,78660
39	CYP1B1	1545	64,4002	36,0841	– 0,835702	0,00642248	1,78473
40	NCOA7	135112	27,7387	15,6627	– 0,824561	0,00642248	1,77100

Note: FPKM1 and FPKM2 indicate fragments per kilobase of exon per million mapped reads after and before PAX8 silencing, respectively. Genes from 1 to 20 were downregulated and genes from 21 to 40 were upregulated.

*CDH6*, a member of the cadherin superfamily, is required for the kidney development as well as ganglia formation [50, 51]. Increased expression of this gene may be associated with tumor growth and metastasis [52]. *CHRD* functions as a dorsalizing factor for early vertebrate embryonic tissues by binding to ventralizing TGF-beta family bone morphogenetic proteins (BMPs) and sequestering them in latent complexes [53]. *ZBED2* (Zinc Finger, BED-Type Containing 2) is a member of family factors involved in the regulation of various functions in vertebrates [54]. *CNTN4* is a glycosylphosphatidylinositol (GPI)-anchored neuronal membrane protein responsible for the formation of axon connections in the developing nervous system [55]. Loss of heterozygosity (LOH) and gene sequencing analysis investigate the possibility of CNTN4 to function as tumor suppressor gene in ovarian cancer [56]. *ANXA2* is a calcium-binding cytoskeletal protein aberrantly expressed in a wide spectrum of cancers and in EOC may promote cell proliferation [57].

Additionally, in siPAX8-FT194 cells among the top 20 significantly upregulated genes there are: *SERPINB2, FPR1, PAPPA, CCL20, RHOB, DCN* (Table 2).

*SERPINB2*, also named Plasminogen Activator Inhibitor, Type II (PAI-2) is upregulated by numerous inflammatory conditions [58]; in cancer disease its expression is often an indicator of positive prognosis as described in ovarian cancer [59, 60]. *FPR1* is a G protein-coupled receptor that promotes growth, angiogenesis and invasion in glioblastoma tumor [61]. FPR1 as the other two FPRs play a pivotal role in inflammatory response, tissue repair, tumor growth, physiological and pathological angiogenesis [62]. *PAPPA* (pregnancy-associated plasma protein A) regulates mitotic progression through modulating the IGF1 signaling pathway [63]. *CCL20* is a small cytokine constitutively produced by Fallopian tube epithelial cells and able to function as endogenous anti-viral microbicide of female reproductive tract [64]. In ovarian cancer, CCL20 is one of the primary chemokine induced via NF-kB pathway [65]. *RHOB*, is a member of small GTPases belonging to the Ras protein superfamily, might have a suppressive activity in cancer progression, in fact its loss occurs frequently in ovary carcinogenesis and progression. Moreover, ectopic expression of RhoB into nude mice is highly effective in suppressing tumor growth of ovarian cancer xenografts [66]. *DCN* is a small leucine-rich proteoglycan, component of connective tissue responsible for the matrix assembly. Also this factor might function as a tumor suppressor in ovarian cancer, because its expression is lost during epithelial transformation [67].

### PAX8 regulates the expression of shared and exclusive sets of genes in SKOV-3 and FT194 cells

**Figure 2:**
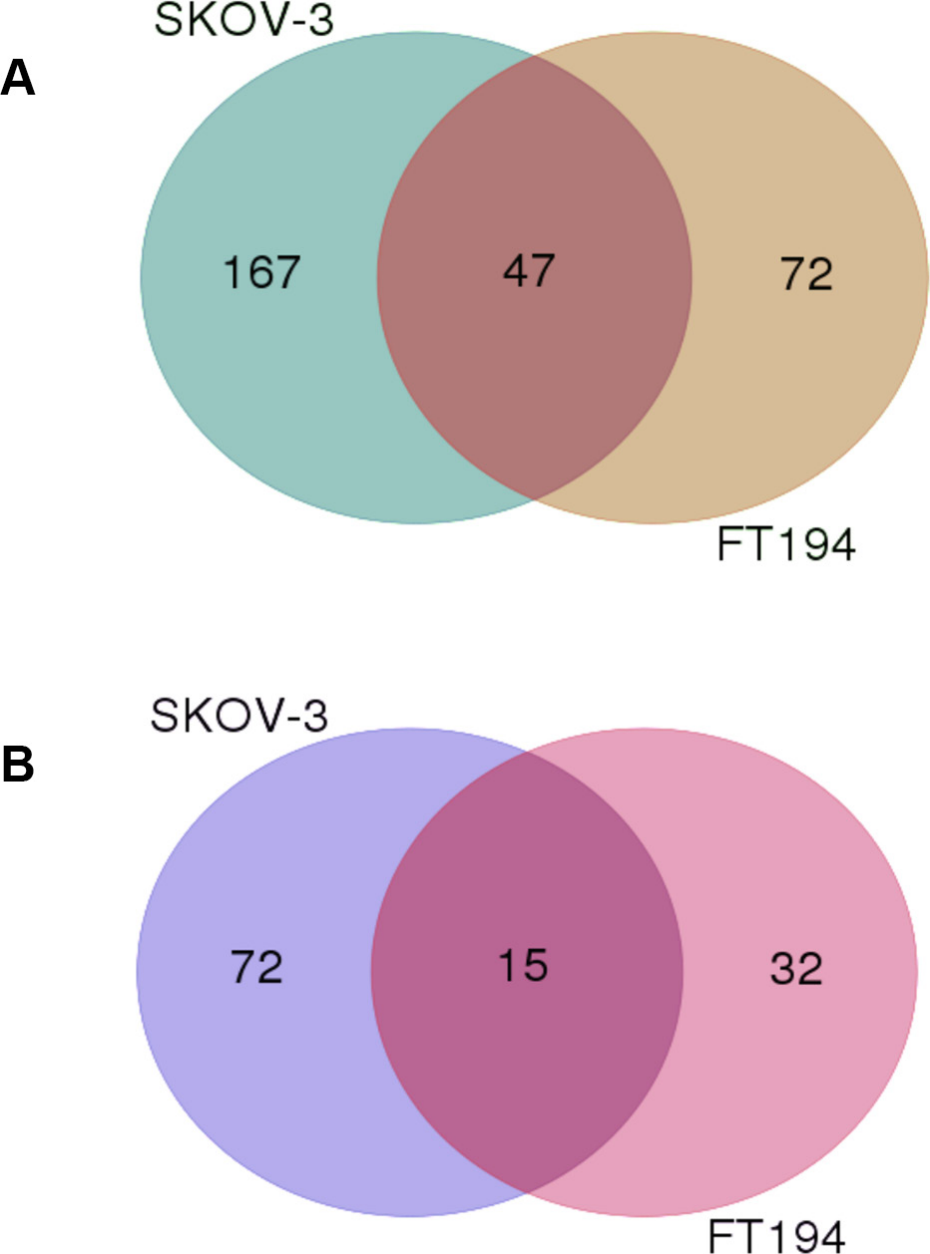
The Venn diagram of genes modulated upon PAX8 knockdown in SKOV-3 and FT194 cells (**A**) Overlap and differences of downregulated genes following PAX8 silencing between SKOV-3 and FT194 cells. (**B**) Overlap and differences of upregulated genes following PAX8 silencing between SKOV-3 and FT194 cells.

As illustrated in the above paragraph, following PAX8 silencing 214 downregulated and 87 upregulated genes were identified in siPAX8-SKOV-3 cells while 119 downregulated and 47 up-regulated were defined in siPAX8-FT194 cells (FDR-adjusted *p*-value ≤ 0.05) (Supplementary Table S3 and Supplementary Table S4). A Venn diagram was constructed to identify shared and distinct sets of genes regulated by PAX8 in the two cell types. As shown in Figure 2A, 47 genes are commonly downregulated in siPAX8-SKOV-3 and siPAX8-FT194 cells, whereas the genes downregulated only in one of the samples are 167 and 72 (SKOV-3 and FT194, respectively). Analogously, 15 genes are commonly upregulated in siPAX8-SKOV-3 and siPAX8-FT194 cells while 72 and 32 genes are exclusively upregulated in SKOV-3 and FT194 cells, respectively (Figure 2B). The complete lists of the genes classified according to the Venn diagram are shown in Table 3.

**Table 3A:** List of shared and exclusive genes downregulated upon PAX8 silencing in SKOV-3 and FT194 cells

Name	Total	Elements
SKOV3-FT194	47	NUP35 ANXA2 HDGFRP3 STX3 DUSP11 AP1G1 ENPP4 PHLDA3 PSAP ENPP1 EPRS RHPN2 C10orf46 GPR63 CDK2AP1 H2AFY2 PPME1 LMLN KITLG CDH6^a^FAM107B C1orf186 BLCAP TEX30 LONRF1 DIAPH1 FGF18^a^ TCN2 ROR1 AKR1B1 TGOLN2 KAZN PHTF2 ARL4C MAL ADCY9 PPAP2B PAX8^a^ ARL5A MAL2 MRE11A HTATSF1 GPR56 PTGIS ANXA2P2 ABCC4 SLC35B4
SKOV3	167	ACTB TSPAN1 RAB12 OSBPL11 L1CAM OSBPL10 LRRC20 RRAD ANKRD34B RNF38 HES2 SMC5 FAM3C PRKAA2 KCNH3 LMAN1 ST3GAL2 AFF1 TAF4B IFNGR1 FHDC1 CGNL1 CA13 SYPL1 MKNK2 SLC39A10 AMMECR1 ZNF114 GAS2L3 CERK BAG4 SASH1 C6orf228 TBC1D13 C7orf29,LRRC61 TUBB2B GTF2E1 ABCC3 PLCH1 CLDND1 MBP CYB5R4 SLC39A14 AKR1B10 CELSR2 PDZK1 FOXN3 PIK3R1 GRIN2B CIAPIN1 CORO1C RNFT1 UPP1 SHROOM2 FAM45A,FAM45B ANKRD33B ZNF702P ID4 PPM1B PER1 SLC44A1 CDH16 PCDH20 ADAM10 RNF130 CBS KIF12^a^KLHL15 ZNF28 ATP8B2 EGFR MIR4723,TMEM199 DICER1 DPP4 ITGB3 DENND1B SGK2 BASP1P1 USP18 RAB3D DCUN1D4 SNTB1 PGGT1B CNKSR3 CHST15 MCAM TYW1 GCH1 TRIM2 C12orf23 FNDC5 IGFN1 USP2 TERF1 SORT1 CRYAB GMCL1 WNT7A^a^ MXRA5 AGL RNF144B ASRGL1 MPHOSPH6 QPRT AATF LIN7C NXT2 C2orf72 RRM2 BCAT1 SLC7A11 ADAMTS9 TMSB4X TPK1 KPNA6 ATP8A1 CHST2 THBS1 CYP4F11 RNF145 ADAMTS5 FLJ26245 SYTL2 UBE2H DCAF12L1 RAB11FIP2 ABI2 PTPRB FIGN PAFAH1B2 TCF12 PMAIP1 WDR44 DCUN1D3 SLC30A6 EPHA4 NEBL DSC2 CNOT6 ZNF611 MYO10 MAP2 KCNS3 SLC26A2 HDHD2 SLAIN1 PLXNC1 MGST1 NUPL1 COL12A1 KCTD5 RCBTB2 CANT1 MTPN FN1 UBE2D1 ZCCHC24 TMTC2 TRIM24 FAM174B C10orf26 TMCO7 GUCY1B3 ARHGEF1 ANKRD52 RC3H2 ZNF618
FT194	72	KLHL13 ZNF185 PPARGC1A WASF1 PLAU KLHL14 TLL2 CHRD^a^ CLGN NGFR ARHGEF37 FLG GAS7 TGFBR3 RGS20 ILDR2 SPON1 MMD CD24 AP1M2 TNFSF4 THY1 ADAMTS14 ZBED2^a^ PLCB4 ADAMTS1 LOC643201 NOV MPP7 SHISA2 ST3GAL1 SOX17 GDF6 ANK3 RASGEF1B CNTN4 C13orf15 SULF2 ADORA1 MEGF9 KLRG2 ITGB8 DANCR,SNORA26 SLC6A6 BAALC CTHRC1 G0S2 TMEM117 AIF1L GJB2 PRKAG2 FAT2 MST1 DCDC2 OXTR SDC2 INHBB KIAA1456 BTBD11 CA2 NID2 SLC47A1 CDH5 PDE1A COL13A1 IGFBP5 TMEM170B CTGF EPHB1 GPRC5C DKK1 CDKN1C

a Genes that were validated using qRT-PCR.

**Table 3B:** List of shared and exclusive genes upregulated upon PAX8 silencing in SKOV-3 and FT194 cells

Name	Total	Elements
SKOV3-FT194	15	ANKRD1 F3 G3BP2 TGFB2 MAPK1IP1L TOMM20 MBTPS1 DNMT3B PTPN1 MSRB3 MET RBPJ TCEB3 WDR1 STX12
SKOV3	72	DIO2^b^ ZCCHC3 NTN4 NGRN TRAPPC2 CALU MOK DCBLD1 PTPMT1 FGF1 C1D AHRR,PDCD6 LOC100507412,RN45S PCSK7 CPA4 CIRBP SERPINE1 C6orf120 PODXL CCDC80 DOCK10 PYGO2 P4HA3 MCFD2 DSEL MYPN LPXN CCND3 CHFR MAP2K4 GRWD1 CCBE1 UBE2G1,ZZEF1 BOD1^b^ NCF2 ODZ2 ERRFI1 CCIN SERPINB5 ATG12 CGB8 FLRT2 AIM1 ROS1 C7orf58 ALS2CL FRMD6 KRT5 SSFA2 PIP4K2A SOCS7 NT5E ESYT2 SMAP1 ARTN SCRN1 DKK1 EFEMP1 RFK SGK1 CCDC68 CRK FOXD1 PGM2L1 PCDH10 SEMA7A ZFAND3 IFFO2 CCNC RCAN1 KHNYN POLR3F
FT194	32	CMPK2 RAD54L2 DDX58 IFIT1 SP110 PTPN13 DCN PAPPA^b^ RHOB CCL20 GBP1 SERPINB2^b^ OAS3 PDZD2 IFIH1 NCOA7 OAS2 RSAD2 PTGS2 MX1 ZCCHC2 CYP1B1 DHX58 DDX60L HMGA2 FARP2 MX2 NRK FPR1 THBD ST6GALNAC5 THBS2

b Genes that were validated using qRT-PCR.

We reasoned that genes modulated upon PAX8 silencing in both cell lines could be looked at as putative PAX8 targets with greater confidence. Hence, we focused our attention on the intersections between SKOV-3 and FT194 cells and we observed that they include several interesting genes such as: *PSAP, FGF18, CDH6, ROR1, RBPJ* and *DNMT3B* whose role in cell proliferation, cell survival and tumorigenic process has already been reported. In particular, PSAP (prosaposin) is a pleiotropic growth factor able to prevent cell death or apoptosis and to promote cell survival [68]; FGF18 controls migration, invasion and tumorigenicity of ovarian cancer cells [69]; CDH6 is a new TGF-beta target modulated as a mesenchymal marker in EMT [52]; ROR1, a receptor tyrosine kinase orphan receptor 1, is involved in migration, invasion and EMT in ovarian cancer cells [70]; RBPJ (recombination signal-binding protein Jk) is a key transcription factor in the Notch signaling pathway and its inhibition reduces cell growth [71]; DNMT3B encodes a DNA methyltransferase involved in de novo DNA methylation whose role in cancer development is not clear but several reports suggest a role as tumor suppressor [72].

At the same time, we believe that genes modulated upon PAX8 silencing exclusively in FT194 or in SKOV-3 cells should be considered relevant because they could reflect the continuous process from the precancerous to the cancerous condition. In addition, the tumorigenic process might itself promote the expression of some genes making them available for PAX8 transcriptional regulation. For example, WNT7A known to function as tumor-promoting in ovarian cancer [73] is present at very low level in FT194 cells but becomes highly expressed in SKOV-3 cells (Supplementary Table S1) where is significantly downregulated upon PAX8 silencing (Supplementary Table S3 and Table 3A). Similarly, ASRGL1 (asparaginase like 1) is highly expressed in ovarian carcinoma and confers a selective growth advantage [74]; accordingly, our data show that ASRGL1 is preferentially expressed in SKOV-3 cells where it is downregulated following PAX8 silencing (Table 3A).

**Figure 3:**
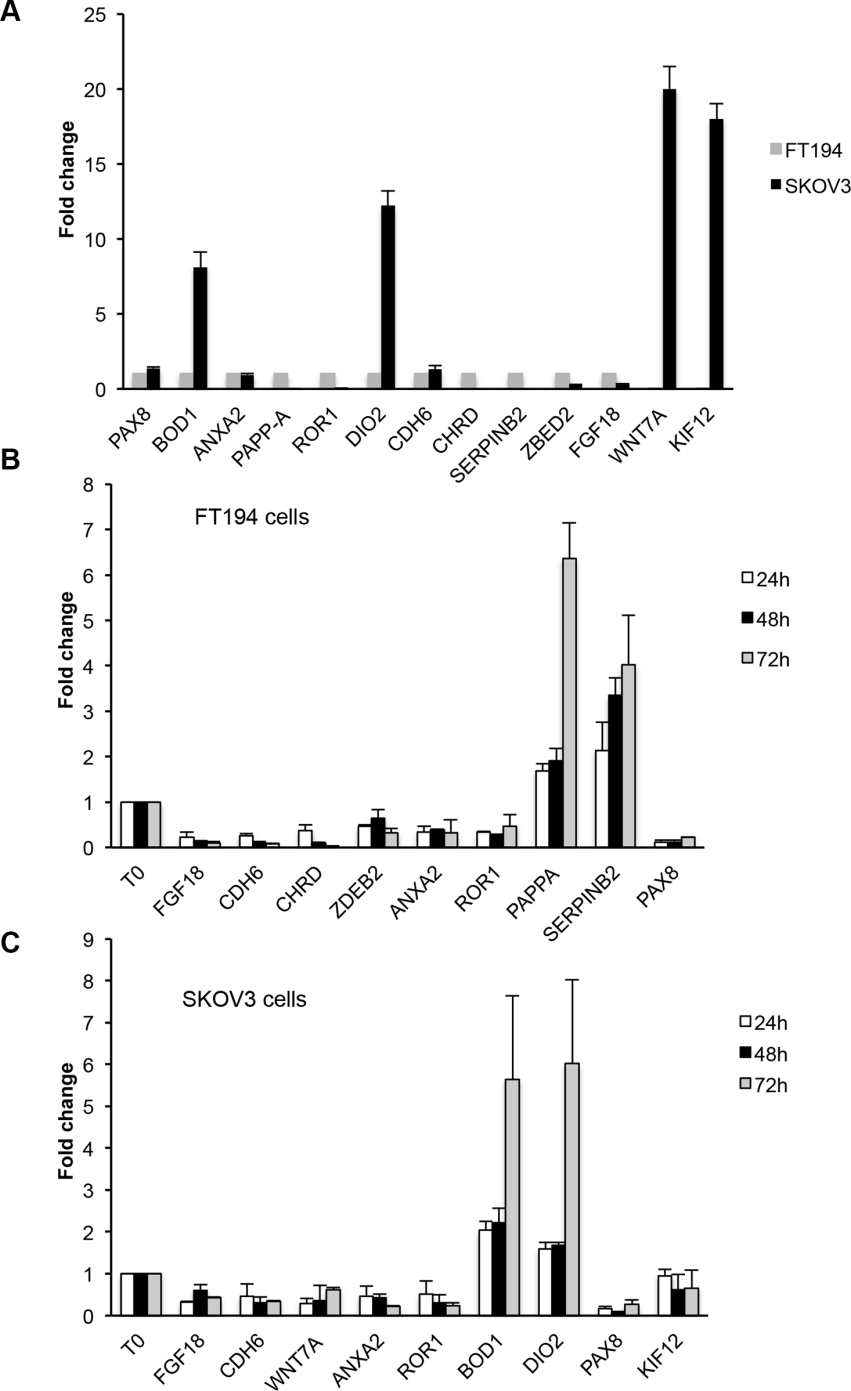
Validation of representative genes by qRT-PCR analysis (**A**) Expression levels of 13 genes measured on total RNA prepared from SKOV-3 and FT194 cells. The values are means ± SD of three independent experiments in duplicate, normalized by the expression of IP08 and expressed as fold change with respect to FT194 cells. (**B** and **C**) Expression levels of some representative genes measured on total RNA prepared from FT194 and SKOV-3 cells transiently transfected with PAX8 siRNA or scramble siRNA 24 h (white bars), 48 h (black bars) and 72 h (grey bars) after transfection. The values are means ± SD of three independent experiments in duplicate, normalized by the expression of IP08 and expressed as fold change with respect to the cells transfected with the scramble siRNA, whose value was set at 1.0. *p*-value was calculated by *t*-test 0.001 ≤ *p* ≤ 0.1.

We validated the data obtained by RNA-seq by means of qRT-PCR for 13 genes, including PAX8. For these genes, we confirmed the different expression between the two cell types (Figure 3A) and in OVCAR-3, PEA1 and PEO14 ovarian cancer cell lines (Supplementary Figure S1). The same genes were also validated upon silencing of PAX8 in FT194 and SKOV-3 cells (Figure 3B and 3C). In particular, we confirmed that KIF12, DIO2 and WNT7A are expressed preferentially in SKOV-3 cells (Figure 3A) and in these cells are modulated upon silencing of PAX8 (Figure 3B). At difference, FGF18, CDH6, ANXA2 and ROR1 are expressed and modulated in both cell lines (Figure 3A, 3B, 3C). CHRD, PAPPA and SERPINB2 are expressed almost exclusively in FT194 cells (Figure 3A) and are modulated upon silencing of PAX8 (Figure 3C). ZBED2 and BOD1 are expressed in both cell lines but following PAX8 silencing ZBED2 is modulated at significant level only in FT194 cells, while BOD1 only in SKOV-3 cells. To strengthen our observations we validated the same genes also in PEA1 cells after PAX8 silencing (Supplementary Figure S2).

**Figure 4:**
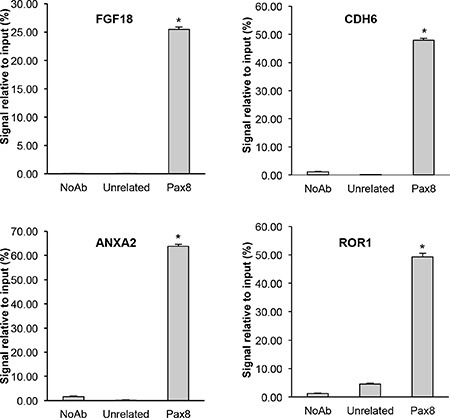
Direct binding of PAX8 on the regulatory regions of putative target genes Chromatin immunoprecipitation assays were performed to determine the binding of PAX8 to the 5′-flanking region of representative genes. Chromatin was subjected to quantitative real-time PCR analysis using appropriate primers (see Materials and Methods). Error bars indicate s.d. between two experiments performed in duplicate (*p* < 0,001).

Subsequently, we analyzed the 5′-flanking regions of the genes commonly regulated in the two cell lines in order to recognize DNA binding motifs for PAX8 matrices using the PASTAA method (http://trap.molgen.mpg.de) [75] which utilizes the prediction of binding affinities of a transcription factor to regulatory regions. The genes containing PAX8 DNA binding motifs were ranked according to the prediction of binding affinity of their 5′-flanking region to the PAX8 binding sites (Supplementary Table S5). To unambiguously determine whether PAX8 directly binds to the above mentioned regulative genomic sequences, we performed a computational analysis for some representative genes chosen among those commonly regulated in the FT194 and SKOV3 cells, using the MatInspector Software (Genomatix). We searched for PAX8 binding sites in a region of about 2 Kb in their 5′-flanking region and we found several PAX8 consensus sequences. To confirm the predictions of the MatInspector analysis, we carried out chromatin immunoprecipitation (ChIP) assays on FT194 cells using a polyclonal antibody against PAX8. The ChIP results indicate that *in vivo* PAX8 is able to bind the regulatory regions of all selected genes (Figure 4).

All together, our results show that the expression profiles affected by PAX8 silencing might reflect an ongoing transformation process reinforcing the involvement of this transcription factor in ovarian cancer.

### Pathways regulated by PAX8 in ovarian cancer

**Figure 5:**
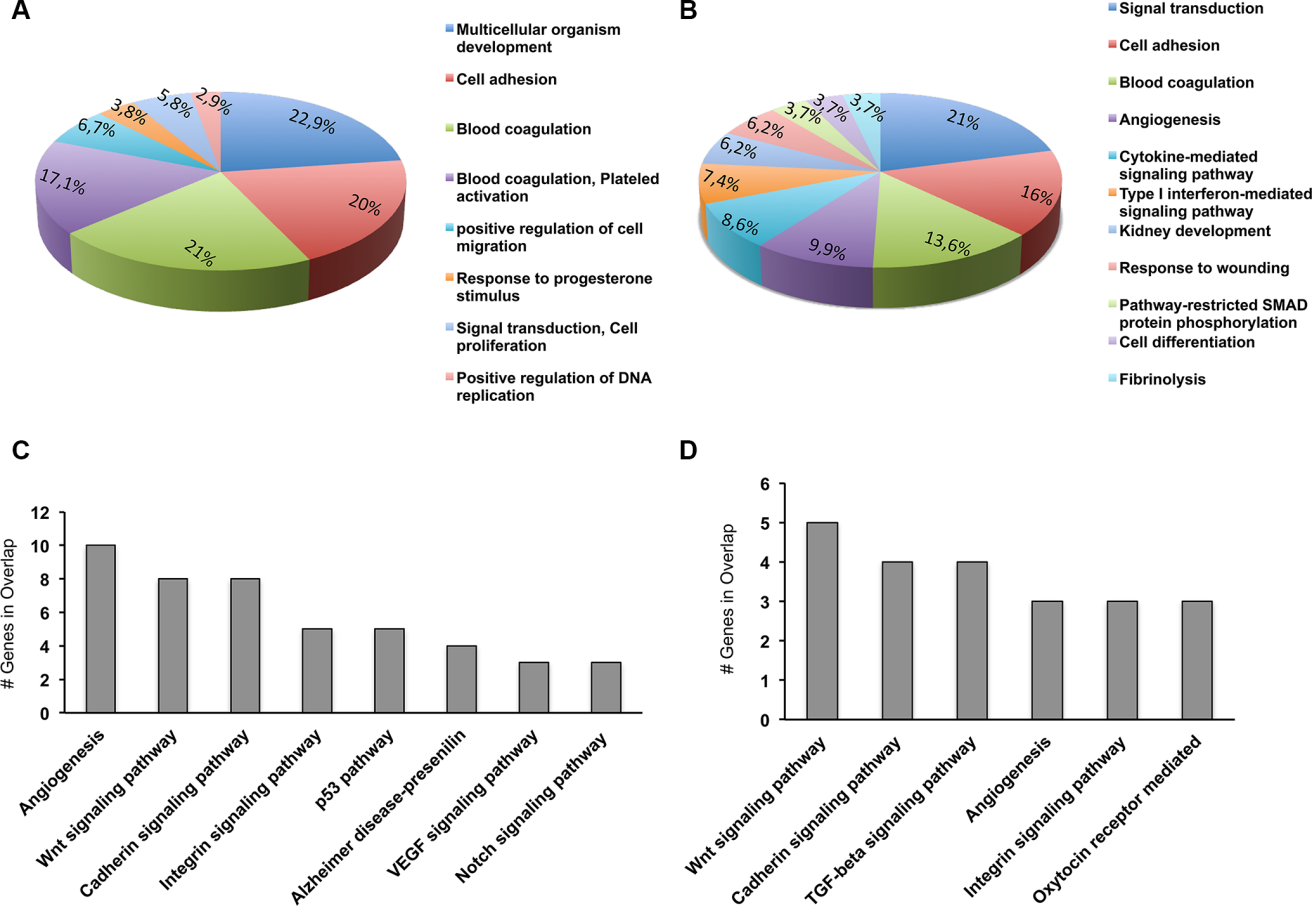
Biological processes and pathways altered in siPAX8 cells Gene ontology (GO) and Panther pathway anlysis have been performed using GeneCodis (http://genecodis.dacya.ucm.es/). (**A**) GO categories enriched for genes modulated upon PAX8 silencing in SKOV-3 cells. (**B**) GO categories enriched for genes modulated upon PAX8 silencing in FT194 cells. (**C**) Panther pathways enriched for genes modulated upon PAX8 silencing in SKOV-3 cells. (**D**) Panther pathways enriched for genes modulated upon PAX8 silencing in FT194 cells.

The pathways that PAX8 may regulate in ovarian cancer and in Fallopian tube secretory cells are still undefined. To categorize PAX8 associated pathways that are represented in our PAX8-silenced SKOV-3 and FT194 cells, we classified all the dysregulated genes (301 for SKOV-3 and 166 for FT194) using the Gene annotations co-occurrence discovery web-based tool (GeneCodis; http://genecodis.dacya.ucm.es/). Several significant GO categories appeared enriched including signal transduction, cell adhesion, blood coagulation, positive regulation of cell migration, angiogenesis and cell differentiation (Figure 5A and 5B). At the same time, the most affected pathways upon silencing of PAX8 comprise Wnt signaling, cadherin signaling, integrin signaling and TGF-beta signaling (Figure 5C and 5D).

**Figure 6:**
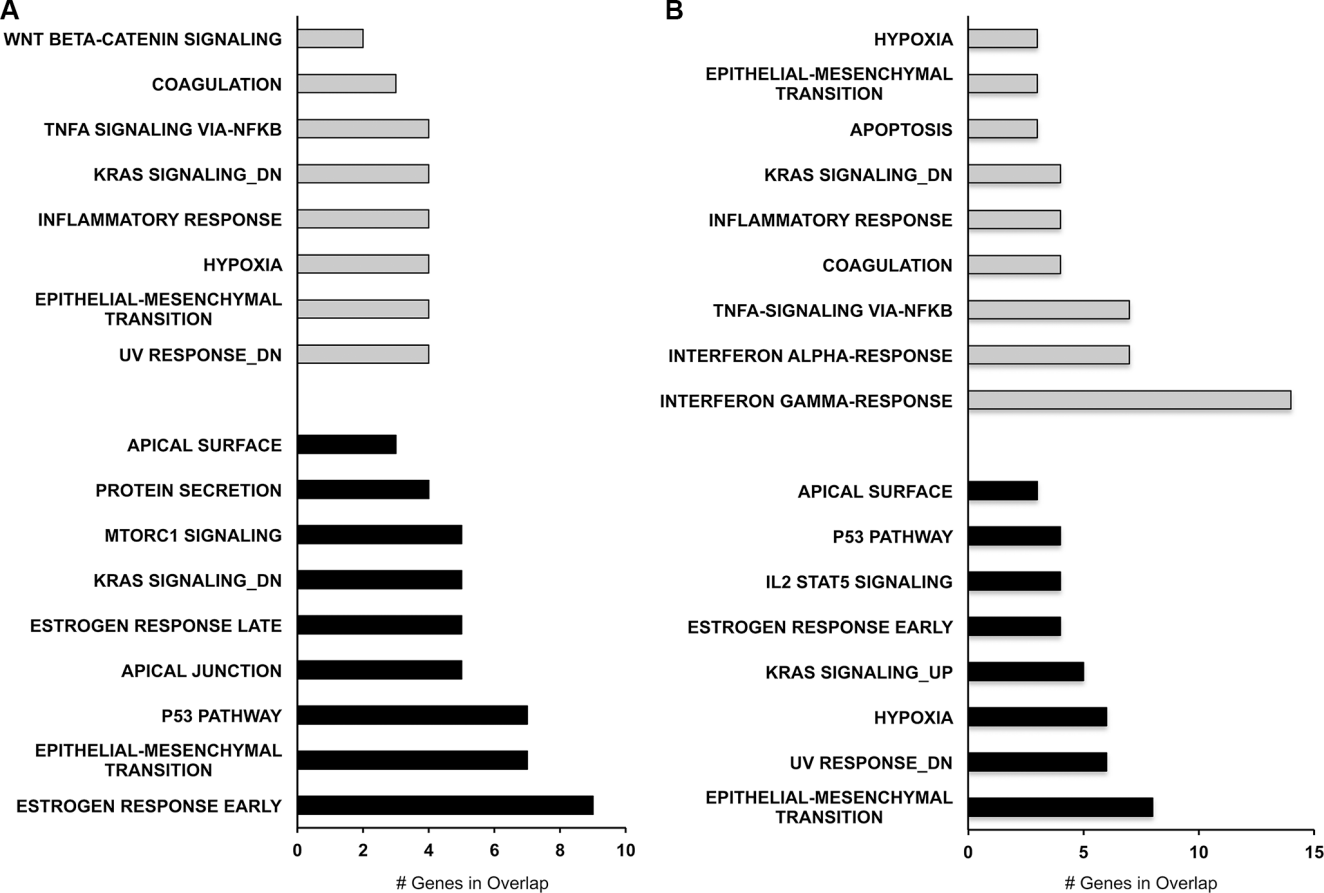
Pathway analysis of differentially expressed genes after PAX8 silencing MsigDB software was used to identify the pathways most affected by the gene dysregulation. (**A**) MsigDB for SKOV-3 downregulated (black bars) and upregulated genes (gray bars). (**B**) MsigDB for FT194 downregulated (black bars) and upregulated genes (gray bars).

To further investigate the functional associations of downregulated and upregulated genes following PAX8 knockdown we performed an MsigDB analysis. Interestingly, we observed that for the significantly upregulated genes (87 for SKOV-3 and 47 for FT194) the most affected pathways in both cells lines are interferon response, TNFa signaling, inflammatory response, apoptosis, UV response and epithelial mesenchymal transition (Figure 6). In the same way, for the downregulated genes (214 for SKOV-3 and 119 for FT194) the most affected pathways in both cells lines are epithelial mesenchymal transition, UV response, Kras signaling, estrogen response and p53 pathway (Figure 6). In agreement with our previous studies, the pathways perturbed upon PAX8 silencing such as IFN-g and TGF-a signaling, EMT, apoptosis, hypoxia as well as UV response strengthen the new role of PAX8 in the regulation of cell survival, proliferation and in the maintenance of oncogenic properties [11, 17].

In conclusion, we think that the majority of the genes affected by PAX8 silencing are associated with important biological cellular processes and we believe that our analysis provides a solid basis for the identification of relevant molecules involved in ovarian cancer.

## DISCUSSION

Recent studies suggest that a substantial proportion of cases of ovarian high-grade serous carcinoma may arise from precursor lesions located in the Fallopian tubal epithelium (FTE). In our study, by means of RNA-seq analysis we investigated the expression of genes modified during the transformation process from Fallopian tube secretory epithelial cells to HGSC cells. Moreover, genes and pathways downstream the transcription factor PAX8 have been analyzed in both cell types. Our goal was to identify new targets for diagnostic and/or therapeutic approaches for HGSC that is the third most common cause of death among gynecologic malignancies worldwide. Actually, improved screening strategies for HGSC diagnosis in early stages, as well as effective treatments are greatly needed.

We highlighted that ≈ 60% of the genes differentially expressed between the two cell types is modulated at a significant level (FDR-adjusted *p*-value ≤ 0.05). Applying a 2 fold-change cutoff and performing a Molecular Signatures Database analysis, we determined in SKOV-3 cells a significant enrichment of estrogen response, EMT, angiogenesis and Wnt/β catenin pathways. At the same time, in FT194 cells we observed an enrichment of pathways like TNF-α, IFN-α, IFN-γ and inflammatory responses. Furthermore, we consider intriguing those genes that turned out to be preferentially, or in some cases exclusively, expressed in FT194 or SKOV-3 cells. There is no doubt that the future challenge will be to examine the potential role of genes differentially expressed between the two cell types as specific and sensitive biomarkers. In fact, several ovarian cancer screening studies have established that acombinatorial biomarker strategy is more reliable, sensitive and specific than using a single protein biomarker [76]. A recent study identified a 5-marker panel for early detection of ovarian cancer that includes five serum biomarkers, namely macrophage-stimulating protein alpha, tissue inhibitor of metalloproteinases-4, platelet-derived growth factor receptor alpha (PDGF-R alpha), osteoprotegerin, and CA-125 [77]. Preliminary insights that we obtained using ProteINSIDE, a novel web service [78], suggest that among the genes highly expressed in SKOV-3 cells there are some that encode for secreted proteins like CGB5 [79], CGB8 [79], FOLR1 [28], SPP1 [80], IGFBP3 [81], NFASC [82] emphasizing that our study may indeed provide a solid basis for the identification of new biomarkers. Of interest, among the top genes highly expressed in SKOV-3 cells there is PRAME that in a wide variety of human malignancies correlates with poor clinical outcome [83]. It has been reported that PRAME proteins are associated to self-renewal cell maintenance [84] and are currently considered as potential target to hamper cancer cell proliferation [85].

In 2003, a gene expression study carried out in ovarian carcinoma [86] reported the transcription factor PAX8, normally absent in ovarian surface epithelial cells, among the most highly expressed genes. The recent carcinogenesis model proposes the secretory cells of the Fallopian tubal mucosa as the cell of origin for the majority of EOC. In agreement with this hypothesis, PAX8 would not be overexpressed in epithelial ovarian carcinoma but rather its expression would be conserved from the cell of origin [38]. To date, the function of PAX8 in Fallopian tube epithelial secretory cells has not been clarified. It has been demonstrated a continually regulated cycle of growth, differentiation, death and renewal in the epithelium of the mammalian oviduct [87] and in this context PAX8 could exert its role. However, more studies are needed to shed light on the function of this transcription factor in this cell type.

We have recently reported that PAX8 is involved in the tumorigenic phenotype of ovarian cancer cells [17]. In the present study, we intend to clarify PAX8 contribution to ovarian cancer through the identification of its downstream gene regulatory network. MsigDB analysis revealed that in both FT194 and SKOV-3 cells the most affected pathways are interferon response, TNFa signaling, inflammatory response, apoptosis, UV response and epithelial mesenchymal transition (considering the upregulated genes) and epithelial mesenchymal transition, UV response, estrogen response and p53 pathway (considering the downregulated genes). Interestingly, our RNA-seq analysis identifies, among PAX8 potential targets, genes that have been reported in the literature having a role in ovarian cancer. One of such genes is FGF18 whose expression is significantly reduced after PAX8 knockdown. It has been demonstrated that FGF18 regulates both tumor cells and tumor microenvironment to facilitate the progression of serous ovarian cancer, enhancing angiogenesis and tumor-associated macrophage infiltration [69, 88]. In addition, FGF18 was identified as the gene possessing the strongest prognostic value in segment 5q31-5q35.3 that was found amplified on microdissected HGSC samples [89]. Furthermore, a recent study provides a platform for the identification of blood-based biomarkers (“Secretome”) for high-grade, advanced-stage serous ovarian tumors and identifies two new markers, FGF18 and GPR172 [90]. Here, we hypothesize that one way in which PAX8 confers a proliferative advantage to ovarian cancer cells is through the regulation of procancer factors, like FGF18, in a pathological microenvironment.

Abnormal activation of the WNT/b-catenin signaling pathway has been associated with ovarian carcinomas. It has been reported that WNT7A is abundantly expressed in ovarian carcinoma and is able to control cell division, adhesion and motility [73]. In addition, WNT7A knockdown cancer cells show a significantly decreased growth rate and invasion ability in a xenografts model [73]. Intriguingly, the phenotype of WNT7A knockdown xenografts resembles that described for the xenograft model of the siPAX8-SKOV-3 cells [17] and this study shows that WNT7A is abundantly expressed only in SKOV-3 cells where is a downstream target of PAX8.

In addition, the expression of some genes regulated by PAX8 in Fallopian tubes secretory cells may be lost during the neoplastic transformation. For example, our data show that chordin (CHRD), a BMP extracellular regulator that behaves as suppressor of tumorigenesis in ovarian carcinoma cells, is abundantly expressed and regulated by PAX8 in FT194 cells, while is almost absent in SKOV-3 cells.

It is worth noting that it has recently been demonstrated that Fallopian tube secretory cell expansion and the ratio between secretory/ciliated cells (S/C ratio) are linked to pelvic serous neoplasia [91] and in this scenario the cancer cells of the serous tubal intraepithelial carcinoma (STIC) bearing a “p53 signature” invade onto the ovary and implant on peritoneal surfaces [92–94]. During this process, the secretory cells retain the expression of PAX8 that possibly continues to exert its transcriptional activity on its physiological targets and in addition may also function on new targets that become available after the tumorigenic hits (BRCA mutations, p53 signature etc).

Our final suggestion is that the relevance of PAX8 in ovarian carcinoma lies in the downstream network(s) regulated by this transcription factor that contribute to ovarian carcinoma pathogenesis. In this respect, we believe that our study has led to the identification of genes and pathways regulated by PAX8 that could be valuable for future studies to uncover the molecular mechanisms leading to EOC.

## MATERIALS AND METHODS

### Cell culture and RNA interference

The human ovarian carcinoma cell line SKOV-3 was obtained from the CEINGE Cell Culture Facility (Naples, Italy) and was grown in RPMI-1640 medium (Euroclone) containing 10% fetal bovine serum (Euroclone). The immortalized Fallopian tube secretory epithelial cell line FT194 was kindly provided by Dr. R. Drapkin (Boston, USA) and was maintained in DME-F12 medium (Euroclone) containing 2% Ultroser G serum (PALL). The OVCAR-3 cell line was obtained from ATCC and was maintained in RPMI-1640 medium (Euroclone) containing 20% fetal bovine serum (Euroclone) and 0.01 mg/ml bovine insulin. PEA1 and PEO14 cells were purchased from Sigma-Aldrich and mantained in RPMI-1640 medium (Euroclone) containing 10% fetal bovine serum (Euroclone), 2 mM glutamine and 2 mM sodium pyruvate (Gibco, Life Technologies).

For RNA interference, SKOV-3, FT194 cells and PEA1 were plated at 2 × 10^5^ cells/60-mm tissue culture dish 24 h prior to transfection and were transfected in replicates with 5 nM PAX8 siRNA (Ambion, Life Techonologies, siRNA ID s15403) or siRNANon-Targeting (Ambion, Life Technologies, siRNA ID 4390843) as scramble, using the Lipofectamine RNAiMAX transfection reagent (Invitrogen) following the manufacturer's protocol. Cells were harvested 24 h after transfection and the total RNA was prepared. Human Fallopian tubes RNA was from Origene (CR559726).

### RNA extraction, qRT–PCR, RNA-seq and data mining

For both qRT-PCR and RNA-seq experiments, total RNA was extracted using the RNeasy Mini kit (Qiagen). For qRT-PCR, the cDNA was synthesized using the iScript cDNA Synthesis kit (BIORAD, Hercules, CA). Real time RT–PCR analysis was performed using the IQ^™^ SYBR Green PCR Master Mix (BIORAD) in a CFX96 Real-Time PCR Detection System (BIORAD) with gene-specific oligonucleotides (Supplementary Table S6).

For RNA-seq, 2 μg of total RNA extracted 24 h after transfection were sent to the Genomics4Life Company (University of Salerno, Italy). Independent silencing experiments were performed from which biological replicates of each condition (siPAX8 or control) were processed for the RNA-seq analysis.

The extracted RNA samples were sequenced using the Illumina HiSeq 1500 platform, TruSeq Stranded Total RNA, 100 bp paired-end reads at the Genomics4Life Company (University of Salerno, Italy).

Analysis was performed using the EPIGEN project sequence facility, RAP (RNA-Seq Analysis Pipeline) [95], available on the following website https://bioinformatics.cineca.it/. Sequence quality was assessed using FastQC (http://www.bioinformatics.babraham.ac.uk/projects/fastqc/) and NGS QC Toolkit [96], using default parameters (PHRED quality score: 20 and for percentage of read length of given quality: 70). Hence, paired-end reads were mapped to the reference human genome build (hg19/GRCh37) using Tophat 2.0.12 [97] with default parameters. The resulting alignment files are provided to Cufflinks [98] to generate a transcriptome assembly and to estimate the expression level expressed as units of FPKM (Fragment mapped per kilobase of exons per million mapped reads). Differential expression analyses were performed with Cuffdiff2 [99] using default parameters. An alpha level of 0.05 was used for all statistical tests. Gene expression data have been submitted to the Gene Expression Omnibus (GEO), Accession number GSE79572.

### Chromatin immunoprecipitation assay

ChIP was performed as previously described [100]. Precleared chromatin from FT194 cells was incubated with 3 μg of affinity-purified rabbit polyclonal anti-PAX8 antibody (Thermo Scientific, PA1-112) or polyclonal anti-TAZ antibody as unrelated (Santa Cruz Biotechnology, sc-17130) and rotated at 4°C for 16 h. Thereafter, the immunoprecipitated DNAs were amplified by quantitative real-time PCR with the following primers:

FGF18 for. 5′-gtgggtagccagtcaagagg-3′; rev. 5′-ctccccaagaacgcagttag-3′;

ANXA2 for. 5′-Gctaaacggctgcaagaaac-3′; rev. 5′-Cgtagcaggcagtcctgag-3′;

CDH6 for. 5′-Atccaacagtggctgactcc-3′; rev. 5′-tctggaaagttgccgaagtt-3′;

ROR1 for. 5′-CAGATCACAGCTGCCTTCAC-3′; rev. 5′-ATTTCACATTCATCGCGACA-3′.

### Pathway analysis

Gene ontology (GO) and Panther pathway analysis have been performed using the GeneCodis tool (http://genecodis.cnb.csic.es) previously described in references [101–103].

Genes showing a two-fold change in expression were analyzed at Molecular Signatures Database (http://software.broadinstitute.org/gsea/msigdb) using the “Hallmark” gene set.

## SUPPLEMENTARY FIGURES AND TABLES










