# Supplementary material for: Candidate genes and pathways downstream of PAX8 involved in ovarian high-grade serous carcinoma
Source: Oncotarget. 2016 May 31;7(27):41929–47. doi: 10.18632/oncotarget.9740 (PMC5173106; doi:10.18632/oncotarget.9740)
Supplement: Supplementary file 1 [file oncotarget-07-41929-s001.pdf]

Candidate genes and pathways downstream of PAX8 involved in ovarian high-grade serous carcinoma

Supplementary Materials

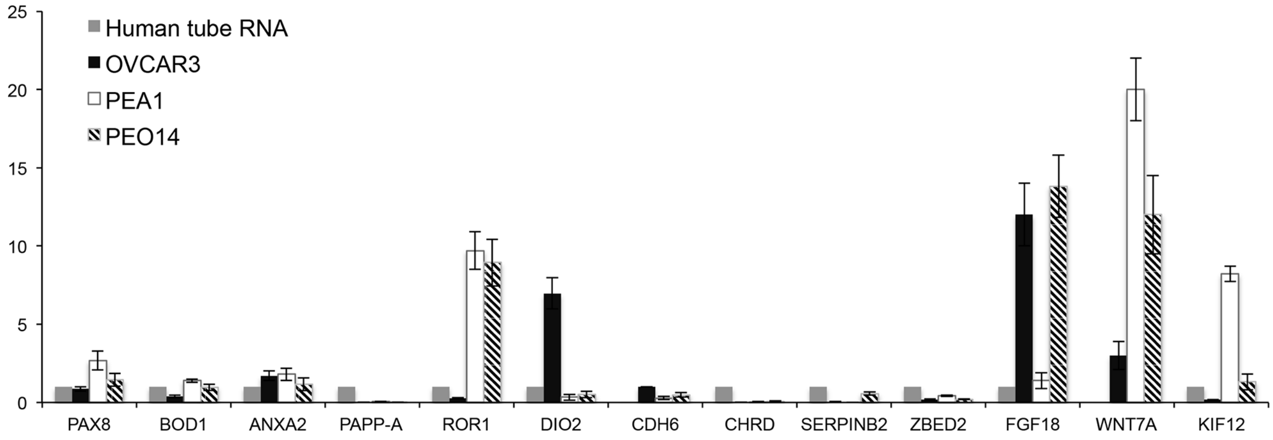

Supplementary Figure S1: Expression levels of 13 genes measured on total RNA prepared from human Fallopian tubes, OVCAR3, PEA1 and PE014 cells. The values are means ± SD of three independent experiments in duplicate, normalized by the expression of IP08 and expressed as fold change with respect to human Fallopian tubes.

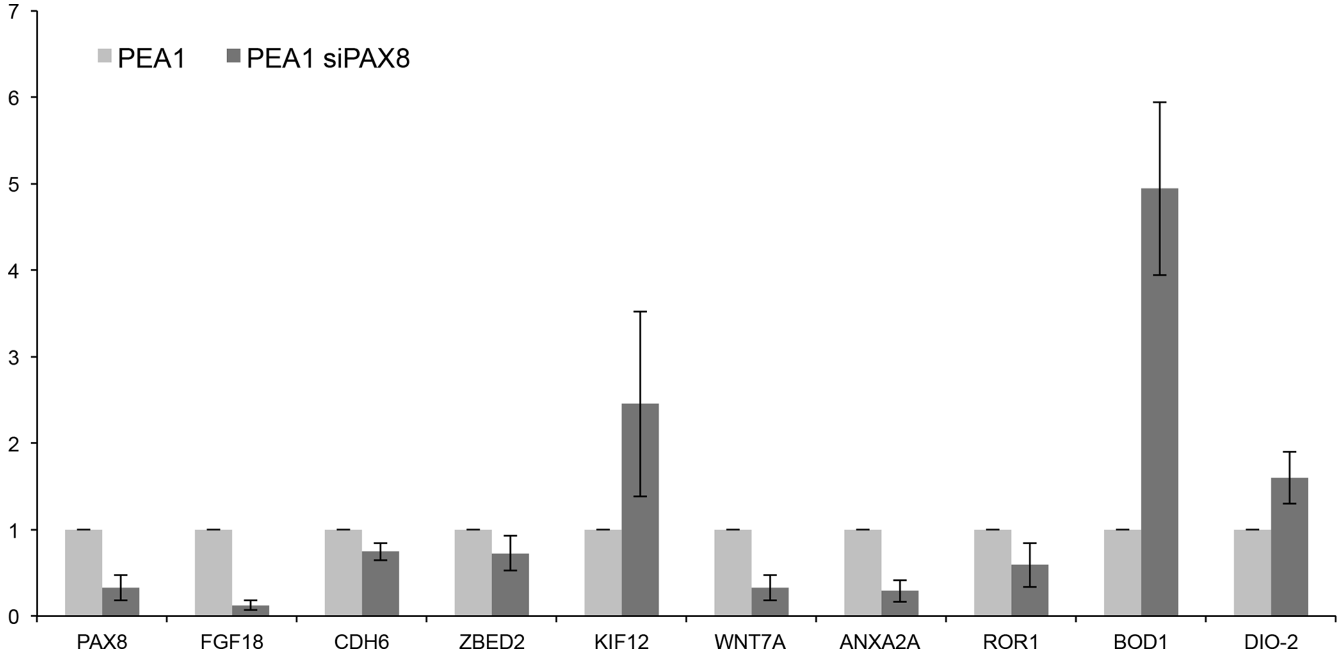

Supplementary Figure S2: Expression levels of some representative genes measured on total RNA prepared from PEA1 cells 24 h after transient transfection with PAX8 siRNA or scramble siRNA. The values are means ± SD of three independent experiments in duplicate, normalized by the expression of IP08 and expressed as fold change with respect to the cells transfected with the scramble siRNA, whose value was set at 1.0.

Supplementary Table S1: Genes expressed in SKOV-3 (FPKM1) and FT194 cells (FPKM2)

Supplementary Table S2: Genes expressed in SKOV-3 (FPKM1) and FT194 cells (FPKM2) at significant level (FDR-adjusted *p*-value ≤ 0.05)

Supplementary Table S3: Genes modulated following PAX8 silencing

Supplementary Table S4: Genes modulated following PAX8 knock-down in FT194 cells

Supplementary Table S5: PASTAA analysis of PAX8 putative target genes

Gene ID	Alternate Gene Synonym	Affinity Score
ENSG00000099284	H2AFY2	2,345
ENSG00000020922	MRE11A	2.28
ENSG00000049130	KITLG	2,144
ENSG00000125257	ABCC4	2,135
ENSG00000197746	PSAP	1,952
ENSG00000131504	DIAPH1	1,833
ENSG00000124212	PTGIS	1,788
ENSG00000156427	FGF18	1,785
ENSG00000174307	PHLDA3	1,744
ENSG00000172005	MAL	1,734
ENSG00000166619	BLCAP	1,714
ENSG00000001561	ENPP4	1,668
ENSG00000125618	PAX8	1,603
ENSG00000136628	EPRS	1,593
ENSG00000006576	PHTF2	1,592
ENSG00000102241	HTATSF1	1,582
ENSG00000182718	ANXA2	1,562
ENSG00000162104	ADCY9	1,543
ENSG00000163002	NUP35	1,511
ENSG00000111328	CDK2AP1	1,497
ENSG00000131941	RHPN2	1,495

ENSG00000185483	ROR1	1,487
ENSG00000085662	AKR1B1	1,473
ENSG00000147676	MAL2	1,464
ENSG00000113361	CDH6	1,461
ENSG00000162407	PPAP2B	1,451
ENSG00000185621	LMLN	1,421
ENSG00000144048	DUSP11	1,418
ENSG00000185339	TCN2	1,377
ENSG00000112218	GPR63	1,374
ENSG00000166747	AP1G1	1,288
ENSG00000197594	ENPP1	1,277
ENSG00000152291	TGOLN2	1,236

Supplementary Table S6: Primers used in qRT-PCR

GENE NAME	FORWARD PRIMER 5′-3′	REVERSE PRIMER 5′-3′
**BOD1**	GCTTACCAAAACCTGAGGCA	CCTGTCTACTCCAGCTTCCA
**DIO2**	CCCCAAGTTGCTTTGCTCAA	AGCAAGTCTACGCTGAGGAT
**PTGS2**	TCCCTTCCTTCGAAATGCAA	TCATCAGGCACAGGAGGAAG
**SERPINB2**	AACCCCAGGCAGTAGACTTC	TCCCCATCTACAGAACCTTCAG
**FGF18**	GGGCAAGGAGACGGAATTCT	AACCTTCTCGATGAACACACAC
**WNT7A**	CAATGGCCGCTGGAACTG	GCAATGATGGCGTAGGTGAA
**KIF 12**	GCTTCTATGTGGAGCAGCTG	ACGGCTGATGTAAAGGGTGA
**CDH6**	ACAGTATTCTACAGGGACAGCC	CACTTGGTACTGCTCCCTGT
**ZBED2**	GACGAGGAAGAGGAGGGAAC	CTTGTTGTGGGGCATTGGAG
**CHRD**	CAGGAGTGGGGGACTAACC	CAGCACCTCAGCAAAGCCT
**ANXA2**	GTTCACGAAATCCTGTGCAA	TGGTCTTGATGGCTGTTTCA
**PAX8**	CCCTTCCAACACGCCACT	CTGCTTTATGGCGAAGGGTG
**IPO8**	AATTCCCAGTACGACAGGCAG	TCACGTATTTGCTGGCGATC
